# Socio-Ecological Predictors of Frequent Bike Share Trips: Do Purposes Matter?

**DOI:** 10.3390/ijerph17207640

**Published:** 2020-10-20

**Authors:** Li-Ting Chen, Ya-Wen Hsu

**Affiliations:** 1 Counseling and Educational Psychology, College of Education, University of Nevada, Reno, NV 89557, USA; litingc@unr.edu; 2 Department of Hospital and Health Care Administration, Chia Nan University of Pharmacy and Science, Tainan 71710, Taiwan

**Keywords:** bikesharing, trip purpose, cycling, active transportation, community health, physical activity

## Abstract

Using bike share could increase physical activity and improve health. This study used the social-ecological model to identify predictors of frequent bike share trips for different purposes. Participants residing in the U.S. were recruited via Amazon Mechanical Turk (MTurk). Self-report trip purposes were used to group participants into using bike share for commuting only (*n* = 260), social/entertainment only (*n* = 313), exercise only (*n* = 358), dual or triple-purpose (*n* = 501), and purposes other than commuting, social/entertainment, and exercise (*n* = 279). Results showed that at the intrapersonal level, perceived use of bike share to be helpful for increasing physical activity was a significant predictor for all groups, except for the other purpose group. Adjusting outdoor activity based on air quality was a significant predictor for the dual or triple-purpose group. At the interpersonal level, having four or more friends/family using bike share was a significant predictor for the other purpose group. At the community level, distance to the nearest bike share within acceptable range was a significant predictor for social/entertainment and dual or triple-purpose groups. The findings suggest that it is important to consider factors at multiple levels for predicting bike share usage. Moreover, health educators and policy makers should adopt different strategies for promoting bike share usage based on trip purposes.

## 1. Introduction

Bike share systems are among the world’s fastest growing mode of public transportation and the number of cities offering bike share has increased in the last decade [1]. According to a report of the National Association of City Transportation Officials, people in the United States took 136 million trips on shared bikes and scooters in 2019, a 60% increase from 2018 [2]. Bike share systems are public services that provide short-term rentals of bicycles. Literature suggests four generations to describe the global evolution of bike share [3,4]. The first generation consisted of free bike systems. Free bike systems were characterized by unlocked and free-of-charge bicycles. In addition, bicycles were placed arbitrarily in a certain regions. The second generation was based on a coin-deposit system. In the second generation, bicycles were locked in docking stations and bicycles were unlocked with coin deposits. Coin deposits were refunded on bicycle returns. The third generation was made up of docked information technology (IT)-based systems, in which bicycles were locked in specific docking stations and users could pay as members or nonmembers. Mobile phones, mag-stripe cards or smartcards could be used for bicycle check-in and check-out. At present, it is the fourth generation—docked and dockless IT-based systems. The characteristics of this generation include an improved locking mechanism to deter bicycle theft, options for electric bicycles, and increased access to bike share through the introduction of dockless bike share. 

In the United States, the top five docked bike share systems are Blue Bikes (Boston), Bay Wheels (San Francisco), Capital Bikeshare (Washington, D.C.), Divvy (Chicago), and Citi Bike (New York) [2,5]. Dockless bike share systems were brought to the United States in 2017 [6]. In 2019, there were 103 docked bike share systems and 71 dockless bike share systems in the United States [6]. Based on data available from Bureau of Transportation Statistics, 85 cities (e.g., Long Beach, Indianapolis, Houston, New York City) had a docked bike share system, 44 cities (e.g., Sacramento, Baltimore, Knoxville, Orlando) had dockless bike share systems, and 15 cities (e.g., Los Angeles, Chicago, Washington, D.C., Tampa) had both docked and dockless bike share systems in 2019 [7]. 

Bike share systems have various potential benefits including increased mobility options, cost savings, lower implementation and operational costs, reduced traffic congestion, reduced fuel use, increased use of public transit and alternative modes, greater environmental awareness, and increased health benefits [3,4,8,9,10,11]. The World Health Organization (WHO) recommends at least 150 min of moderate-intensity aerobic physical activity throughout the week for adults aged 18–64 [12]. Based on the results of the 2018 National Health Interview Survey, approximately 53% adults in the United States aged 18 and over met the guidelines [13]. Previous research has shown that active transportation, such as cycling, is associated with increased physical activity and better health [14,15]. Moreover, increased physical activity is related to lower risks of chronic disease [16]. It was estimated that in developed countries, 1% of the whole population might use bike share, of which two-thirds will already meet the WHO physical activity requirements [8]. Therefore, if the bike share users who did not meet the minimum requirements of physical activity met the requirements through using bike share, it would be a 0.33% increase on the prevalence of population who meet the physical activity recommendation [8]. Yet with a proliferation of bike share programs, 0.33% may underestimate the potential impact of bike share programs on a population meeting physical activity guidelines. By comparing the health benefits and risks of using bike share, studies conducted in Europe revealed that the health benefits of physical activity outweighed the health risk of traffic fatalities and air pollution [9,10]. 

In order to change health behavior, the social-ecological model specifies that factors at multiple levels should be considered. These levels consist of intrapersonal, interpersonal, organizational, community, and public policy levels [17]. The model has been used to understand the multiple determinants of health behaviors, such as accessing health services among sex workers [18], the linkage between poverty and smoking [19], and cycling among older adults [20]. In terms of predicting cycling behavior, intrapersonal factors may include perceived self-efficacy for cycling, general health, air quality consciousness, and socio-demographic characteristics [21,22,23,24]. Interpersonal factors may include a family’s decision to cycle together, participation in a cycling club, and simply having family or friends who engage in cycling [21,23,25]. At the organizational level, a company may implement a workplace health promotion program encouraging the employees to engage in cycling [26]. Community factors may include built environment characteristics, such as street connectivity and traffic safety [21,22,27]. Public policy factors may include bike-transit integration and land use [28]. 

Cycling for different purposes has been found to be associated with different enabling factors and barriers [24,27,29,30]. A survey conducted in Vancouver, Canada, examined the effects of safety concern, energy consciousness, and air quality consciousness on cycling frequency for cycling for commuting, shopping/errands/dining, and recreation/exercise [24]. Results showed that energy consciousness was associated with more cycling for recreation/exercise but not for commuting or shopping/errands/dining. In addition, air quality consciousness was associated with more cycling for shopping/errands/dining and recreation/exercise but not for commuting. One study conducted in the United States with participants in Texas and Alabama showed that perceived environment factors had stronger associations with transportation cycling than with recreation-only cycling [29]. Another review article included 39 studies published between 2007 and 2017 to examine the associations between 12 built environment factors (e.g., street/route connectivity and land use mix) and cycling among people who cycled for transportation, commuting, recreation, and general cycling [30]. For people who cycled for transportation, large effect sizes were found for street connectivity and access to nonresidential destinations. For people who cycled for commuting, both street connectivity and cycling facilities/paths had large effects on cycling. For people who cycled for general cycling, large effect sizes were identified for open space/green space, aesthetics/attractiveness, and cycling facilities/cycling paths. Yet, none of the 12 built environmental factors had a large effect on cycling for people who cycled for recreation. 

Similar to research focused on cycling behavior, it is important to understand trip purposes for using bike share [1]. Although the socio-ecological model has been used to investigate enabling factors and barriers of cycling behavior, few studies have used this model to examine bike share usage. Research conducted in Washington, D.C., with bike share users and regular cyclists showed that bike share users were more likely to be female and younger, and to have lower household income than regular cyclists [31]. Predictors for bike share usage may likewise differ from predictors for cycling. Hence, this study aims to identify socio-ecological predictors of frequent bike share trips for users with different purposes. 

We identified trip purposes for using bike share through prior research. Commuting (travel to/from work or school) and social/entertainment were the most common trip purposes in a survey conducted with people in Minneapolis–St. Paul and Washington, D.C., in the United States [32]. While using bike share has the potential benefits of physical activity, the predictors of bike share usage for exercise or fitness particularly may be different from users who do not use bike share primarily for exercise. In addition to using bike share for commuting and social/entertainment, using bike share for exercise was included in this study. Lastly, factors associated with frequent bike share trips for users who use bike share for one primary purpose may be different from users who use bike share for dual or triple-purpose. Therefore, the present study focuses on identifying socio-ecological predictors of frequent bike share trips for commuting only, social/entertainment only, exercise only, dual or triple-purpose from commuting, social/entertainment, and exercise, and purposes other than commuting, social/entertainment, and exercise. To our knowledge, no study has applied the socio-ecological model to examine predictors of bike share usage for these five purposes. 

The remainder of this paper is organized as follows. We describe the data, the socio-ecological predictors, and the analysis strategies in Section 2. Results of the analyses for each of the five groups are presented in Section 3. In Section 4 and Section 5, we present discussions and conclude this study, respectively. 

## 2. Materials and Methods 

### 2.1. Participants

Participants were recruited from Amazon Mechanical Turk (MTurk), an online crowdsourcing platform, from May to September 2019. MTurk allows researchers to collect a large amount of quality data quickly and for relatively little cost [33,34]. In MTurk, requesters are people who post or request tasks (e.g., surveys) to be completed, whereas workers are people who are paid for task completion. Requesters can customize the tasks to be available to certain MTurk workers. MTurk workers are able to read descriptions of tasks and select the tasks they are interested in. A report published in 2019 estimated that there were 250,810 workers worldwide and more than 226,500 of these workers were based in the United States [35]. 

In this study, we restricted the visibility of the survey to MTurk workers who resided in the United States. Participants in this study were MTurk workers who self-selected to participate in the study. After they consented to participate in this study, they were screened for eligibility. Four criteria were used to determine eligible participants: (1) being at least age 20, (2) currently residing in a city in the United States with bike share systems, (3) using bike share within one year, and (4) not having a medical condition that limited exercise capacity. If eligible, participants completed the survey via Qualtrics and then received $2.50 via MTurk for compensation. The protocol (1422942-1) was approved by the Institutional Review Board at the University of Nevada, Reno. 

### 2.2. Measures

#### 2.2.1. Bike Share Trips for Different Purposes

**Figure 1:**
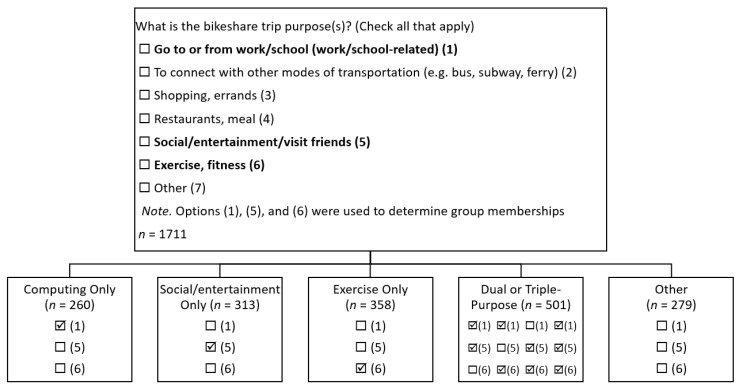
The method for grouping participants of five bike share trip purposes; Bold options were used to divide participants into five groups.

A total of 1711 eligible participants were included in the study. Participants were categorized into five groups based on the purposes of their bike share trips: (I) commuting only, (II) social/entertainment only, (III) exercise only, (IV) dual or triple-purpose from commuting, social/entertainment, and exercise, and (V) purposes other than commuting, social/entertainment, and exercise. Specifically, participants were asked to choose their bike share trip purposes. Options for trip purposes included (1) to go to or from work/school (work/school-related), (2) to connect with other modes of transportation (e.g., bus, subway, and ferry), (3) for shopping/errands, (4) for restaurants/meal, (5) for social/entertainment/visiting friends, (6) for exercise or fitness, and (7) other. Figure 1 presents the method for grouping participants of five bike share trip purposes. 

Participants who chose (1) but chose neither (5) nor (6) for their bike share trip purposes were grouped as using bike share for commuting only (*n* = 260). Participants who chose (5) but chose neither (1) nor (6) were grouped as social/entertainment only (*n* = 313). Participants who chose (6) but chose neither (1) nor (5) were grouped as exercise only (*n* = 358). Participants who chose two or three from (1), (5), and (6) were grouped as dual or triple-purpose (*n* = 501). Among these 501 participants, 37 chose (1) and (5), 78 chose (1) and (6), 314 chose (5) and (6), and 72 chose all three. 

Participants who chose none from (1), (5), and (6) for their bike share trip purposes were grouped as purposes other than commuting, social/entertainment, or exercise, or simply other (*n* = 279). Among these 279 participants, 93 chose their bike share purposes for “to connect with other modes of transportation,” 155 chose their bike share purposes for “shopping, errands,” 60 chose their bike share purposes for “restaurants, meal,” and 17 chose their bike share purposes for “other.” 

#### 2.2.2. Socio-Demographic Variables

Participants were asked to report on several socio-demographic variables, including gender, age, race, height, weight, the highest level of education completed, household’s yearly income, having/caring for children younger than 16 or not, marital status, employment status, and state they resided in.

#### 2.2.3. Socio-Ecological Predictors

**Figure 2:**
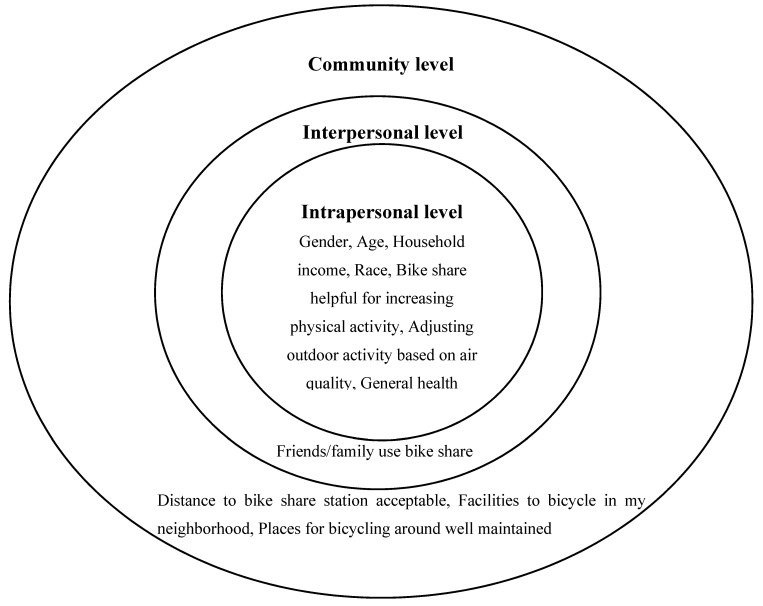
Socio-ecological predictors of frequent bike share trips; Bold fonts indicate the three levels of the socio-ecological model examined in this study.

Literature suggests intrapersonal variables (e.g., biological, psychological), social environment (e.g., social model), and physical environment (e.g., bicycle infrastructure) should be considered in studying physical activity [17,36]. Therefore, this study focuses on the socio-ecological predictors from the three inner levels of the socio-ecological model: intrapersonal level, interpersonal level, and community level (Figure 2). 

At the intrapersonal (Intra) level, participants were asked: “Intra-1. How helpful do you think using the bike share system is in increasing your physical activity?” “Intra-2. How often do you check the level of air quality in your city through any media channels (e.g., news, internet)?” and “Intra-3. Would you say your health in general is?” Participants were asked to answer Intra-1 using a 4-point scale (1 = not at all helpful, 2 = slightly helpful, 3 = moderately helpful, 4 = very helpful). Participants’ responses to Intra-1 were further grouped into three categories: not at all helpful/slightly helpful, moderately helpful, and very helpful. Not at all helpful/slightly helpful was used as the reference group for predicting frequent bike share trips. Participants were asked to answer Intra-2 using a 4-point scale (1 = rarely, 2 = sometimes, 3 = often, 4 = always). Participants’ responses to Intra-2 were further grouped into two groups: rarely (reference) and sometimes to always. Participants were asked to answer Intra-3 using a 5-point scale (1 = poor, 2 = fair, 3 = good, 4 = very good, 5 = excellent). Participants’ responses to Intra-3 were further grouped into three categories: poor/fair (reference), good, and very good/excellent. In addition to these three predictors, gender (male as the reference), age (20–29 years old as the reference), household’s yearly income (≤$39,999 as the reference), and race (Caucasian as the reference) were also the predictors of frequent bike share usage at the intrapersonal level. As a result, there were seven predictors at the intrapersonal level. 

At the interpersonal (Inter) level, participants were asked: “Inter-1. How many of your friends/family currently use the bike share system?” Participants were asked to answer Inter-1 using a 5-point scale (0 = 0, 1 = 1–3, 2 = 4–6, 3 = 7–9, 4 = ≥10). Participants’ responses to Inter-1 were further grouped into three categories: 0 (reference), 1–3, and ≥4 friends/family currently use the bike share system. 

At the community (Cmnt) level, participants were asked to respond to four questions: “Cmnt-1. In general, how long would it take to get from your home/work/school to the nearest bike share station, if you walked there?” “Cmnt-2. To you, what is the acceptable distance from your home/work/school to the nearest bike share station?” “Cmnt-3. There are facilities to bicycle in or near my neighborhood, such as special lanes, separate paths or trails, shared use paths for cycles and pedestrians. Would you say that you?” and “Cmnt-4. Places for bicycling (such as bike paths) in and around my neighborhood are well maintained and not obstructed. Would you say that you?” For both Cmnt-1 and Cmnt-2, participants were asked to answer the questions using a 4-point scale (1 = ≤10 min, 2 = 11–20 min, 3 = 21–30 min, 4 = >30 min). Cmnt-1 and Cmnt-2 were further used to determine if the nearest bike share station was within acceptable distance for a participant. For example, if a participant chose “11–20 min” for Cmnt-2, the participant would be grouped as within acceptable distance to the nearest bike share station if the participant also chose “≤10 min” or “11–20 min” for Cmnt-1. In contrast, if a participant chose “11–20 min” for Cmnt-2, the participant would be grouped as beyond acceptable distance to the nearest bike share station if the participant also chose “21–30 min” or “>30 min” for Cmnt-1. Cmnt-3 and Cmnt-4 were the two bicycling infrastructure items from the 17-item self-reported measure of Physical Activity Neighborhood Environment Scale [37]. Participants were asked to answer these two items using a 4-point scale (1 = strongly disagree, 2 = somewhat disagree, 3 = somewhat agree, 4 = strongly agree). Participants’ responses to Cmnt-3 and Cmnt-4 were both converted to binary variables with two values: disagree (reference) and agree. Therefore, there were three predictors at the community level. 

#### 2.2.4. Dependent Measure 

The dependent measure was a binary variable: whether or not the participant made two or more trips per week in the past six months. The question “In the past 6 months, how many bike share trips did you make per week?” was used to create the binary variable. Participants were asked to answer this question using a 4-point scale (1 = ≤1 trip per week, 2 = 2–5 trips per week, 3 = 6–9 trips per week, 4 = ≥10 trips per week). Participants’ responses were further grouped into two categories: one or less bike share trip (reference) and two or more bike share trips per week in the past six months. 

### 2.3. Analyses 

Descriptive statistics were performed to examine the characteristics of participants in the five groups (i.e., using bike share for commuting only, social/entertainment only, exercise only, dual or triple-purpose, and other). Within each group, a chi-square test was performed to evaluate if the responses for each socio-demographic characteristic were even across response options for bike share users. To determine the socio-ecological predictors of frequent bike share usage, binary logistic regression was performed separately for users with the five purposes. All the analyses were performed in IBM SPSS Statistics v. 26.0 (IBM corp, Armonk, NY, USA). An alpha level of 0.05 was used for statistical significance. Results of logistic regression are presented as odds ratios (ORs) with 95% confidence intervals (CIs) and *p*-values for the predictors. The cutoff value of 0.5 for predicted probability was used for correct classification rates. When the predicted probability was equal to or greater than 0.5, the participant was predicted to make two or more bike share trips per week. The participant was predicted to make one or less bike share trip per week otherwise. The correct classification rate for making one or less bike share trip per week was calculated from the number of participants who were correctly predicted to make one or less bike share trip divided by the number of participants who actually reported one or less bike share trip per week. Correct classification rate for making two or more bike share trips per week was calculated from the number of participants who were correctly predicted to make two or more bike share trips divided by the number of participants who actually reported two or more bike share trips per week. Overall correct classification rate was calculated from the number of correctly predicted participants divided by the total number of the participants. 

## 3. Results

Table 1 presents descriptive statistics and results of chi-square tests. Findings showed that for all five groups, there were more male and younger participants. The majority of participants were Caucasian, had at least a 4-year college degree, were under/normal weight, did not have/care for children younger than 16 years old, were employed full-time, and lived in the South. Three of the five groups, namely the commuting, social/entertainment, and dual or triple-purpose groups, had significant results on household’s yearly income. Specifically, the commuting group had a higher frequency on household’s yearly income ≤$39,999 than the expected value and lower frequencies than the expected values on household’s yearly income $60,000–$79,999 and ≥$80,000. Both the social/entertainment and dual or triple-purpose groups had higher frequencies on household’s yearly income ≤$39,999, $40,000–$59,999, and ≥$80,000 than their expected values and a lower frequency on household’s yearly income $60,000–$79,999 than the expected value. In terms of marital status, four groups, namely the social/entertainment, exercise, dual or triple-purpose, and other groups, had higher frequencies of participants never having been married, higher frequencies of participants being married, and lower frequencies of participants being in the other category than their corresponding expected values. The commuting group had a higher frequency of participants that had never been married than the expected value and lower frequencies of participants that were married and were in the other category than their expected values. The sections that follow present results of binary logistic regression for users with the five purposes. 

### 3.1. Bike Share Trips for Commuting Only

Among participants who reported commuting only as their bike share trip purposes, 75 participants (29.4%) made one or less bike share trip per week and 180 participants (70.6%) made two or more bike share trips per week in the past six months. Five participants had missing data on some variables and were not included in the analyses of logistic regression. Results of predicting making two or more bike share trips per week for commuting only are presented in Table 2. The Negelkerke *R*^2^ for the model was 0.31. Correct classification rates were 48.0% for making one or less bike share trip per week and 91.7% for making two or more bike share trips per week. The overall correct classification rate was 78.8%. 

Compared to Caucasians, African Americans were more likely to make two or more trips per week (OR = 6.17; 95% CI = 1.30, 29.23). Those who believed that using bike share system is moderately helpful for increasing physical activity were more likely to make two or more trips per week than those who believed using bike share system is not at all or slightly helpful for increasing physical activity (OR = 4.73; 95% CI = 1.98, 11.34). Furthermore, those who believed that using bike share system is very helpful for increasing physical activity were more likely to make two or more trips per week than those who believed using bike share system is not at all or slightly helpful for increasing physical activity (OR = 9.01; 95% CI = 3.74, 21.72). 

### 3.2. Bike Share Trips for Social/Entertainment Only

Among participants who reported social/entertainment only as their bike share trip purposes, 203 participants (66.3%) made one or less bike share trip per week and 103 participants (33.7%) made two or more bike share trips per week in the past six months. Seven participants had missing data on some variables and were not included in the analyses of logistic regression. Results for predicting making two or more bike share trips per week for social/entertainment are presented in Table 2. The Negelkerke *R*^2^ for the model was 0.27. Correct classification rates were 87.7% for making one or less bike share trip per week and 40.8% for making two or more bike share trips per week. The overall correct classification rate was 71.9%. 

Compared to participants whose yearly household income were ≤$39,999, those whose yearly household income were $60,000–$79,999 were less likely to make two or more trips per week (OR = 0.40, 95% CI = 0.16, 0.99). Those who believed that using bike share system is moderately helpful for increasing physical activity were more likely to make two or more trips per week than those who believed using bike share system is not at all or slightly helpful for increasing physical activity (OR = 3.71; 95% CI = 1.80, 7.64). Furthermore, those who believed that using bike share system is very helpful for increasing physical activity were more likely to make two or more trips per week than those who believed using bike share system is not at all or slightly helpful for increasing physical activity (OR = 5.68; 95% CI = 2.62, 12.33). When the nearest bike share station was within the acceptable distance from one’s home/work/school, participants were more likely to make two or more trips per week (OR = 2.18; 95% CI = 1.20, 3.99). 

### 3.3. Bike Share Trips for Exercise Only 

Among participants who reported exercise only as their bike share trip purposes, 164 participants (49.0%) made one or less bike share trip per week and 171 participants (51.0%) made two or more bike share trips per week in the past six months. Twenty-three participants had missing data on some variables and were not included in the analyses of logistic regression. Results for predicting making two or more bike share trips per for exercise are presented in Table 2. The Negelkerke *R*^2^ for the model was 0.19. Correct classification rates were 65.9% for making one or less bike share trip per week and 63.7% for making two or more bike share trips per week. The overall correct classification rate was 64.8%. 

Compared to participants who were between 20 and 29 years old, those who were between 30 and 39 years old were less likely to make two or more trips per week (OR = 0.47; 95% CI = 0.27, 0.84). Similarly, those who were at least 40 years old were less likely to make two or more trips per week than those who were between 20 and 29 years old (OR = 0.39; 95% CI = 0.21, 0.75). Compared to Caucasians, Hispanics were more likely to make two or more bike share trips per week (OR = 2.96; 95% CI = 1.20, 7.30). Those who believed that using bike share system is very helpful for increasing physical activity were more likely to make two or more trips per week than those who believed using bike share system is not at all or slightly helpful for increasing physical activity (OR = 2.75; 95% CI = 1.28, 5.93). 

### 3.4. Bike Share Trips for Dual or Triple-Purpose from Commuting, Social/Entertainment, and Exercise

Among participants who reported dual or triple-purpose from commuting, social/entertainment, and exercise, 202 participants (41.3%) made one or less bike share trip per week and 287 participants (58.7%) made two or more bike share trips per week in the past six months. Twelve participants had missing data on some variables and were not included in the analyses of logistic regression. Results for predicting making two or more bike share trips per week for dual or triple-purpose are presented in Table 3. The Negelkerke *R*^2^ for the model was 0.22. Correct classification rates were 46.0% for making one or less bike share trip per week and 83.6% for making two or more bike share trips per week. The overall correct classification rate was 68.1%. 

Compared to Caucasians, African Americans were more likely to make two or more bike share trips per week (OR = 2.17; 95% CI = 1.07, 4.41). Those who believed that using bike share system is moderately helpful for increasing physical activity were more likely to make two or more trips per week than those who believed using bike share system is not at all or slightly helpful for increasing physical activity (OR = 2.87; 95% CI = 1.42, 5.81). Furthermore, those who believed that using bike share system is very helpful for increasing physical activity were more likely to make two or more trips per week than those who believed using bike share system is not at all or slightly helpful for increasing physical activity (OR = 5.17; 95% CI = 2.62, 10.20). Adjusting outdoor activity based on air quality was also associated with two or more bike share trips per week (OR = 1.67; 95% CI = 1.10, 2.52). When the nearest bike share station was within the acceptable distance from one’s home/work/school, participants were more likely to make two or more trips per week (OR = 1.77; 95% CI = 1.15, 2.74). 

### 3.5. Bike Share Trips for Purposes Other than Commuting, Social/Entertainment, and Exercise 

Among participants who did not select commuting, social/entertainment, or exercise as their bike share purposes, 137 participants (50.0%) made one or less bike share trip per week and 137 participants (50.0%) made two or more bike share trips per week in the past six months. Five participants had missing data on some variables and were not included in the analyses of logistic regression. Results for predicting making two or more bike share trips per week with users who did not select commuting, social/entertainment, or exercise as their bike share purposes are presented in Table 3. The Negelkerke *R*^2^ for the model was 0.29. Correct classification rates were 70.8% for making one or less bike share trip per week and 61.3% for making two or more bike share trips per week. The overall correct classification rate was 66.1%. 

Compared to Caucasians, African Americans were more likely to make two or more trips per week (OR = 4.03; 95% CI = 1.41, 11.51). Those who had four or more friends/family currently use the bike share system were more likely to make two or more trips per week than those who had 0 (OR = 8.30; 95% CI = 2.89, 23.88). 

## 4. Discussion

### 4.1. Summary of Results and Practical Implications

Bike share has the potential to improve health, and people worldwide are making an increasing number of bike share trips [1,2,8,14,15]. In this study, we applied the social-ecological model and included seven predictors at the intrapersonal level, one predictor at the interpersonal level, and three predictors at the community level to predict frequent bike share trips for five groups of users who used bike share for different purposes. These five groups were people who used bike share for commuting only, social/entertainment only, exercise only, dual or triple-purpose from commuting, social/entertainment, and exercise, and purposes other than commuting, social/entertainment, and exercise. Our results highlighted the importance of considering trip purposes for promoting bike share usage, which was consistent with conclusions drawn from prior research on cycling [24,27,29,30].

Similar to previous research [38,39,40], the majority of bike share users in this study were young, male, and Caucasian. In addition, most of them had at least a 4-year college education and were full-time employees [32,38]. When gender, age, household income, race, perceived bike share as helpful for increasing physical activity, adjusting outdoor activity based on air quality, and general health were included in the logistic regression model as the intrapersonal level predictors of frequent bike share usage, along with other predictors at the interpersonal and community levels, gender was not a significant predictor for any group. Being younger was associated with frequent bike share usage only for the exercise only group. Household income was a significant predictor only for the social/entertainment group. Lastly, African Americans were more likely to make frequent bike share trips than Caucasians for commuting, dual or triple-purpose, and other purposes. Hispanics were more likely to make frequent bike share trips than Caucasians for exercise. 

One study conducted in the Netherlands showed that people who cycled to work considered it important that their mode of commuting was healthy [41]. In addition, an awareness of environmental benefits, health benefits, and mental relaxation stimulated cycling for a long commute [41]. Studies comparing the benefits and risks of using bike share showed that the health benefits of physical activity outweighed the health risk of traffic fatalities and air pollution [9,10]. Our findings provided additional evidence that perceived use of bike share to be helpful for increasing physical activity could be used to predict frequent bike share trips. This implies that health educators and policy makers may design programs that emphasize the positive effects of bike share on physical activity to promote bike share usage. 

Another study conducted in Vancouver, Canada, showed that air pollution consciousness was higher in those with higher cycling frequency [24]. In this study, adjusting outdoor activity based on air quality was found to be associated with frequent bike share trips for dual or triple-purpose. Regarding the impact of particulate matter (PM) levels on bike share, one study conducted in Seoul, South Korea, revealed that PM_10_ and PM_2.5_ levels had negative impact on bike share use, particularly in winter and spring [42]. Another study reviewed seven studies on air quality and physical activity and concluded that poor air quality was related to lower odds of physical activity [43]. It is possible that people who worry about the harmful effects of air pollution have a higher level of awareness toward air quality. Thus, they may make decisions to cycle outdoors after making sure the air quality is good. Integrating air quality index in the bike share rental platforms may encourage these people to use bike share. 

At the interpersonal level, our findings showed that having four or more friends/family use bike share was associated with frequent bike share trips for the other purposes group but not for the remaining four groups. The result implies that when people use bike share for purposes other than commuting, social/entertainment, and exercise, the frequency of their bike share trips is related to the prevalent usage of bike share among their friends and families. The study conducted in Belgium also found that having significant others to cycle with (accompany effect) or without (modeling effect) could both predict cycling for commute [23]. Indeed, literature suggests one of the keys to a successful bike share is to encourage people to adopt a positive attitude towards these bikes [44]. Research also recommends using multistage behavior change theory to align interventions with user readiness to turning intentions to use bike share into actions [45]. 

Significant relationships between built environment factors and cycling behavior have been found [29,30]. Our results revealed that bicycling infrastructure was not significantly related to frequent bike share trips for any of the five groups. The inconsistencies may be related to the inclusion of variables at the intrapersonal and interpersonal levels along with bicycling infrastructure. Yet, perceived distance to bike share station within acceptable range was the significant predictor for social/entertainment and dual or triple-purpose groups. This implies the accessibility of bike share services is vital for users who use bike share for these two purposes.

This study was motivated by prior research on examining motivators and barriers to cycling for different purposes. In addition to categorizing bike share users into groups based on trip purposes, studies may look into the heterogeneity of users based on other characteristics, such as users who are in the top 10 percentile of usage among all members [46] or university campus users [47]. This study is a starting point in understanding the differences among bike share users with different purposes. 

### 4.2. Limitations and Future Research Directions 

Participants of the present study were recruited from U.S. MTurk workers who had used any bike share systems within one year. Findings from this study may not generalize to all bike share users. For example, the participants in this study may be more technology savvy than general bike share users. Future studies may collaborate with bike share vendors to recruit a larger and representative sample of bike share users. Future studies may also explore the effect of docked/dockless bike share on transport and health. As dockless bike share systems increase users’ access to service and hold promise for offering equitable access to bike share, it is important to understand the impact of the emergence of dockless bike share services [4,48]. When bike share system data can be linked to users’ survey data, researchers can further extend this study to examine the effects of socio-ecological predictors on travel characteristics, such as travel distance and travel time [44,49]. In addition, the data were collected using online surveys. Findings from the study do not imply causal relationship between the predictors and frequent bike share usage. Future research may use intervention studies to examine the causal relationship between the significant predictors and bike share usage, such as a workshop for increasing physical activity using bike share programs. In addition, future studies may include objective measurements to predict bike share usage, such as air quality index. Lastly, the socio-ecological predictors at the organizational level and the public policy level were not included in this study. Future studies may explore how factors from these two levels affect bike share usage, such as the bicycle safety laws. The possibilities of interaction effects of predictors across levels may also be considered. 

## 5. Conclusions

Our study is one of the very few that adopt the socio-ecological model to identify predictors for bike share usage. We extend bike share research by examining predictors of bike share usage for different trip purposes. Findings from this study add to the limited literature on the association between perceived health benefits and bike share usage. In conclusion, results of this study support the assumption that predictors at multiple levels and trip purposes should be considered for determining bike share usage. Furthermore, in order to promote bike share usage and related health benefits, health educators and policy makers should implement customized strategies for promoting bike share usage based on trip purposes. 

## Figures and Tables

**Table 1:** Characteristics of participants by bike share trip purposes. Significant results are in bold.

Characteristics	Commuting, *n* = 260	Social/Entertainment, *n* = 313	Exercise, *n* = 358	Dual or Triple-Purpose, *n* = 501	Other, *n* = 279
Gender					
Male	164 (63.1%)	172 (55.0%)	199 (55.6%)	292 (58.4%)	160 (57.6%)
Female	96 (36.9%)	141 (45.0%)	159 (44.4%)	208 (41.6%)	118 (42.4%)
Missing	0	0	1	1	1
χ^2^(*p* value)	**17.79 (<0.001)**	**3.07 (0.080)**	**4.47 (0.035)**	**14.11 (<0.001)**	**6.35 (0.012)**
Age					
20–29	116 (44.6%)	118 (37.7%)	108 (30.2%)	211 (42.1%)	102 (36.6%)
30–39	104 (40.0%)	129 (41.2%)	154 (43.0%)	207 (41.3%)	115 (41.2%)
40 and above	40 (15.4%)	66 (21.1%)	96 (26.8%)	83 (16.6%)	62 (22.2%)
Missing	0	0	0	0	0
χ^2^(*p* value)	**38.52 (<0.001)**	**21.71 (<0.001)**	**15.71 (<0.001)**	**63.43 (<0.001)**	**16.41 (<0.001)**
Race					
Caucasian	165 (63.5%)	208 (66.5%)	232 (65.0%)	316 (63.5%)	182 (65.5%)
Hispanic	15 (5.8%)	32 (10.2%)	33 (9.2%)	49 (9.8%)	19 (6.8%)
African American	33 (12.7%)	31 (9.9%)	49 (13.7%)	57 (11.4%)	32 (11.5%)
Other	47 (18.1%)	42 (13.4%)	43 (12.0%)	76 (15.3%)	45 (16.2)
Missing	0	0	1	3	1
χ^2^(*p* value)	**213.05 (<0.001)**	**287.81 (<0.001)**	**305.89 (<0.001)**	**395.831 (<0.001)**	**247.67 (<0.001)**
Education					
<4-year college	92 (35.4%)	117 (37.4%)	121 (33.8%)	174 (34.8%)	85 (30.6%)
≥4-year college	168 (64.6%)	196 (62.6%)	237 (66.2%)	326 (65.2%)	193 (69.4%)
Missing	0	0	1	0	1
χ^2^(*p* value)	**22.22 (<0.001)**	**19.94 (<0.001)**	**37.59 (<0.001)**	**46.21 (<0.001)**	**41.96 (<0.001)**
BMI ^1^					
Under/normal weight	135 (52.5%)	161 (52.3%)	181 (51.9%)	275 (55.9%)	141 (51.8%)
Overweight	67 (26.1%)	94 (30.5%)	113 (32.4%)	153 (31.1%)	80 (29.4%)
Obese	55 (21.4%)	53 (17.2%)	55 (15.8%)	64 (13.0%)	51 (18.8%)
Missing	3	5	9	9	7
χ^2^(*p* value)	**43.46 (<0.001)**	**57.90 (<0.001)**	**68.38 (<0.001)**	**136.84 (<0.001)**	**46.55 (<0.001)**
Household income (USD)					
≤39,999	85 (32.7%)	83 (26.5%)	95 (26.5%)	134 (26.8%)	86 (30.9%)
40,000–59,999	65 (25.0%)	85 (27.2%)	105 (29.3%)	140 (28.0%)	64 (23.0%)
60,000–79,999	48 (18.5%)	52 (16.6%)	79 (22.1%)	96 (19.2%)	62 (22.3%)
≥80,000	62 (23.8%)	93 (29.7%)	79 (22.1%)	130 (26.0%)	66 (23.7%)
Missing	0	0	0	1	1
χ^2^(*p* value)	**10.74 (0.013)**	**12.46 (0.006)**	**5.49 (0.139)**	**9.38 (0.025)**	**5.34 (0.149)**
Children younger than 16 years					
No	195 (75.0%)	218 (69.6%)	218 (60.9%)	324 (64.8%)	173 (62.2%)
Yes	65 (25.0%)	95 (30.4%)	140 (39.1%)	176 (35.2%)	105 (37.8%)
Missing	1	0	0	1	1
χ^2^(*p* value)	**65.00 (<0.001)**	**48.34 (<0.001)**	**16.99 (<0.001)**	**43.81 (<0.001)**	**16.63 (<0.001)**
Marital status					
Never been married	163 (62.7%)	182 (58.1%)	163 (45.5%)	261 (52.2%)	137 (49.3%)
Married	81 (31.2%)	107 (34.2%)	171 (47.8%)	197 (39.4%)	116 (41.7%)
Other ^2^	16 (6.2%)	24 (7.7%)	24 (6.7%)	42 (8.4%)	25 (9.0%)
Missing	0	0	1	0	1
χ^2^(*p* value)	**125.22 (<0.001)**	**119.74 (<0.001)**	**114.51 (<0.001)**	**152.16 (<0.001)**	**76.50 (<0.001)**
Employment status					
Full-time employee	204 (78.5%)	242 (77.3%)	298 (83.2%)	421 (84.0%)	223 (79.9%)
Other	56 (21.5%)	71 (22.7%)	60 (16.8%)	80 (16.0%)	56 (20.1%)
Missing	0	0	0	0	0
χ^2^(*p* value)	**84.25 (<0.001)**	**93.42 (<0.001)**	**158.22 (<0.001)**	**232.10 (<0.001)**	**99.96 (<0.001)**
Region ^3^					
Northeast	53 (20.4%)	64 (20.4%)	65 (18.2%)	96 (19.2%)	54 (19.4%)
Midwest	50 (19.2%)	58 (18.5%)	73 (20.4%)	102 (20.4%)	45 (16.1%)
South	80 (30.8%)	110 (35.1%)	133 (37.2%)	182 (36.3%)	92 (33.0%)
West	77 (29.6%)	81 (25.9%)	87 (24.3%)	121 (24.2%)	88 (31.5%)
Missing	0	0	0	0	0
χ^2^(*p* value)	**11.35 (0.010)**	**20.82 (<0.001)**	**30.96 (<0.001)**	**37.00 (<0.001)**	**24.21 (<0.001)**

^1^ Body mass index (BMI) was calculated as weight/height^2^ and participants were categorized into three groups: under/normal weight (BMI < 25), overweight (25 ≤ BMI < 30), and obese (BMI ≥ 30). ^2^ Other included being widowed, separated, and divorced. ^3^ Reginal definitions were based on the US Census Bureau. Specifically, the Northeast included Maine, New Hampshire, Vermont, Massachusetts, Rhode Island, Connecticut, New York, New Jersey, and Pennsylvania. The Midwest included Ohio, Michigan, Indiana, Wisconsin, Illinois, Minnesota, Iowa, Missouri, North Dakota, South Dakota, Nebraska, and Kansas. The South consisted of Delaware, Maryland, Virginia, West Virginia, Kentucky, North Carolina, South Carolina, Tennessee, Georgia, Florida, Alabama, Mississippi, Arkansas, Louisiana, Texas, Oklahoma, and Washington, D.C. The West consisted of Montana, Idaho, Wyoming, Colorado, New Mexico, Arizona, Utah, Nevada, California, Oregon, Washington, Alaska, and Hawaii.

**Table 2:** Likelihood of making two or more trips per week in the past six months for commuting, social/entertainment, and exercise. Significant predictors are in bold.

Predictors	Two or More Trips for Commuting OR (95% CI)	Two or More Trips for Social/Entertainment OR (95% CI)	Two or More Trips for Exercise OR (95% CI)
Gender (female vs. male)	0.59 (0.29–1.19); 0.138	0.91 (0.53–1.58); 0.742	0.80 (0.49–1.29); 0.356
Age 30–39 vs. 20–29	1.20 (0.59–2.43); 0.614	0.76 (0.41–1.41); 0.379	**0.47 (0.27–0.84); 0.011**
Age ≥40 vs. 20–29	0.59 (0.23–1.54); 0.280	1.08 (0.51–2.30); 0.845	**0.39 (0.21–0.75); 0.004**
Household income $40,000–$59,999 vs. ≤$39,999	1.63 (0.65–4.07); 0.295	0.72 (0.35–1.50); 0.384	1.43 (0.76–2.69); 0.275
Household income $60,000–$79,999 vs. ≤$39,999	0.83 (0.33–2.04); 0.679	**0.40 (0.16–0.99); 0.048**	1.32 (0.66–2.65); 0.428
Household income ≥$80,000 vs. ≤$39,999	1.72 (0.69–4.30); 0.246	0.65 (0.31–1.35); 0.243	1.19 (0.59–2.40); 0.622
Hispanic vs. Caucasian	0.74 (0.20–2.77); 0.655	1.48 (0.63–3.50); 0.373	**2.96 (1.20–7.30); 0.019**
African American vs. Caucasian	**6.17 (1.30–29.23); 0.022**	0.97 (0.40–2.36); 0.948	1.69 (0.84–3.39); 0.143
Other vs. Caucasian	1.22 (0.53–2.79); 0.644	0.66 (0.27–1.59); 0.354	1.13 (0.52–2.47); 0.795
Bike share moderately helpful for increasing physical activity vs. not at all/slightly helpful	**4.73 (1.98–11.34); <0.001**	**3.71 (1.80–7.64); <0.001**	1.88 (0.86–4.12); 0.116
Bike share very helpful for increasing physical activity vs. not at all/slightly helpful	**9.01 (3.74–21.72); <0.001**	**5.68 (2.62–12.33); <0.001**	**2.75 (1.28–5.93); 0.010**
Adjusting outdoor activity based on air quality sometimes to always vs. rarely	0.96 (0.50–1.83); 0.893	1.49 (0.86–2.59); 0.157	1.15 (0.70–1.88); 0.580
General health good vs. poor or fair	0.82 (0.27–2.44); 0.720	1.87 (0.58–6.06); 0.298	1.52 (0.61–3.76); 0.366
General health very good/excellent vs. poor or fair	0.75 (0.25–2.21); 0.602	1.54 (0.48–4.90); 0.466	1.66 (0.69–3.99); 0.256
1–3 friends/family use bike share vs. 0	1.76 (0.81–3.80); 0.150	0.81 (0.24–2.71); 0.734	0.67 (0.34–1.33); 0.252
≥4 friends/family use bike share vs. 0	2.96 (0.85–10.32); 0.088	2.90 (0.82–10.22); 0.098	1.50 (0.63–3.60); 0.362
Distance to bike share station acceptable (acceptable vs. unacceptable)	0.50 (0.24–1.04); 0.063	**2.18 (1.20–3.99); 0.011**	1.38 (0.84–2.27); 0.205
Bicycling around well maintained (agree vs. disagree)	1.09 (0.50–2.35); 0.836	0.59 (0.31–1.15); 0.122	1.43 (0.74–2.76); 0.294
Facilities to bicycle in my neighborhood (agree vs. disagree)	1.90 (0.80–4.52); 0.147	2.08 (0.95–4.54); 0.066	0.82 (0.40–1.66); 0.571

**Table 3:** Likelihood of making two or more trips per week in the past six months for dual or triple-purpose and other purposes. Significant predictors are in bold.

Predictors	Two or More Trips for Dual or Triple-Purpose OR (95% CI)	Two or More Trips for Other Purposes OR (95% CI)
Gender (female vs. male)	0.82 (0.54–1.23); 0.331	1.36 (0.76–2.43); 0.294
Age 30–39 vs. 20–29	1.16 (0.75–1.81); 0.512	0.84 (0.45–1.58); 0.596
Age ≥40 vs. 20–29	0.84 (0.47–1.50); 0.553	0.91 (0.43–1.94); 0.807
Household income $40,000–$59,999 vs. ≤$39,999	1.05 (0.61–1.81); 0.859	2.10 (0.93–4.73); 0.074
Household income $60,000–$79,999 vs. ≤$39,999	1.61 (0.88–2.96); 0.122	1.00 (0.46–2.17); 1.000
Household income ≥$80,000 vs. ≤$39,999	0.96 (0.54–1.69); 0.886	0.93 (0.44–2.00); 0.857
Hispanic vs. Caucasian	1.73 (0.84–3.54); 0.136	0.90 (0.29–2.81); 0.849
African American vs. Caucasian	**2.17 (1.07–4.41); 0.032**	**4.03 (1.41–11.51); 0.009**
Other vs. Caucasian	1.00 (0.56–1.77); 0.989	2.11 (0.96–4.67); 0.064
Bike share moderately helpful for increasing physical activity vs. not at all/slightly helpful	**2.87 (1.42–5.81); 0.003**	1.04 (0.49–2.19); 0.923
Bike share very helpful for increasing physical activity vs. not at all/slightly helpful	**5.17 (2.62–10.20); <0.001**	2.13 (0.96–4.75); 0.063
Adjusting outdoor activity based on air quality sometimes to always vs. rarely	**1.67 (1.10–2.52); 0.015**	1.31 (0.74–2.34); 0.354
General health good vs. poor or fair	1.80 (0.81–4.01); 0.150	1.09 (0.42–2.83); 0.858
General health very good/excellent vs. poor or fair	1.64 (0.75–3.61); 0.217	1.40 (0.58–3.39); 0.454
1–3 friends/family use bike share vs. 0	0.55 (0.24–1.28); 0.165	1.80 (0.88–3.69); 0.109
≥4 friends/family use bike share vs. 0	1.12 (0.46–2.73); 0.808	**8.30 (2.89–23.88); <0.001**
Distance to bike share station acceptable (acceptable vs. unacceptable)	**1.77 (1.15–2.74); 0.010**	1.45 (0.73–2.90); 0.291
Bicycling around well maintained (agree vs. disagree)	0.77 (0.45–1.32); 0.340	1.23 (0.58–2.62); 0.586
Facilities to bicycle in my neighborhood (agree vs. disagree)	1.30 (0.73–2.31); 0.369	1.40 (0.63–3.11); 0.410
